# Qingre Huoxue decoction attenuates myocardial ischemia‒reperfusion injury by regulating the autophagy‒endoplasmic reticulum stress axis via FAM134B-mediated ER-phagy

**DOI:** 10.3389/fphar.2024.1447610

**Published:** 2024-11-18

**Authors:** Rui Li, Jiechun Zhang, Shuliang Ji, Junfeng Fang, Xiaodong Ji, Yanping Zeng, Nan Liu, Wei Wu, Shiyi Liu

**Affiliations:** ^1^ Department of Emergency, The First Affiliated Hospital of Guangzhou University of Chinese Medicine, Guangzhou, Guangdong, China; ^2^ Guangdong Clinical Research Academy of Chinese Medicine, Guangzhou, Guangdong, China; ^3^ Department of Intensive Care Unit, The Affiliated TCM Hospital of Guangzhou Medical University, Guangzhou, Guangdong, China; ^4^ Department of Traditional Chinese Medicine, Shantou University Medical College, Shantou, Guangdong, China; ^5^ Department of Cardiology, The First Affiliated Hospital of Guangzhou University of Chinese Medicine, Guangzhou, Guangdong, China

**Keywords:** Qingre Huoxue decoction, autophagy, endoplasmic reticulum stress, ER-phagy, myocardial ischaemia/reperfusion injury, FAM134B

## Abstract

**Background:**

Autophagy‒endoplasmic reticulum (ER) stress axis dysregulation is linked to myocardial ischemia‒reperfusion injury (MIRI), which counteracts the benefits of acute myocardial infarction (AMI) reperfusion therapy. Qingre Huoxue decoction (QRHX) improves the short- and long-term prognosis of AMI after percutaneous coronary intervention and alleviates myocardial injury in AMI rats by stimulating autophagy via the PI3K/Akt pathway. We aimed to further explore the efficacy of QRHX in treating MIRI and its regulatory relationship with FAM134B-mediated ER-phagy.

**Materials and methods:**

Rats were administered different concentrations of QRHX for 2 weeks, and then MIRI was induced. Ultra-performance liquid chromatography‒tandem mass spectrometry (UPLC‒MS) was used to examine the levels of the main pharmacological metabolites of the serum of rats treated with QRHX. H9c2 cells were pretreated with QRHX-mediating serum (QRHX-MS) for 24 h before being exposed to hypoxia/reoxygenation (H/R). The mechanisms underlying the effects of QRHX-MS were further studied via rescue experiments involving FAM134B knockdown. The myocardial infarct size, cardiac function, morphology and the expression of apoptosis-, autophagy-, and ER stress-related proteins and genes were assessed. The colocalization of autophagosomes with lysosomes and the localization of proteins involved in ER-phagy or autophagic flux was examined.

**Results:**

QRHX decreased the myocardial infarct size and oxidative stress, improved cardiac function and alleviated morphological changes in a dose-dependent manner in MIRI rats by promoting autophagic flux to inhibit ER stress and ER stress-related apoptosis, which was related to FAM134B-mediated ER-phagy, as revealed by autophagy analysis. UPLC‒MS analysis of QRHX-MS revealed 20 major active metabolites of QRHX-MS, including baicalin, cryptotanshinone, 3,4-dihydroxybenzaldehyde and caffeic acid. QRHX-MS attenuated H/R-induced cardiomyocyte injury and apoptosis by increasing autophagic flux to suppress ER stress and ER stress-related apoptotic protein and gene expression. When autophagic flux was inhibited or FAM134B was knocked down in H9c2 cells followed by QRHX-MS pretreatment, the protective effect of QRHX was partially reversed.

**Conclusion:**

QRHX alleviates myocardial injury, apoptosis and infarct size expansion in MIRI by regulating the autophagy‒ER stress axis via FAM134B-mediated ER-phagy.

## 1 Introduction

With the development of percutaneous coronary intervention (PCI), the inpatient mortality of acute myocardial infarction (AMI) in China has decreased to 4.3% (Ma et al., 2021). According to the SWEDEHEART registry, the occurrence of heart failure (HF) as an in-hospital complication of AMI has decreased from 46% during the thrombolytic therapy period to 28% in the era of PCI (Desta et al., 2015). However, the myocardial infarction reperfusion-induced coronary microcirculation injury, myocardial oxidative stress, calcium overload, endoplasmic reticulum stress, autophagy dysregulation, and iron deposition can lead to a second wave of myocardial necrosis, resulting in a larger infarct size than observed during reperfusion, significantly increasing the risk of major adverse cardiovascular events (MACE) (Vora et al., 2024). It is reported that cardiovascular mortality among patients who undergo PCI due to AMI within 1 year is as high as 11% (Heusch, 2020). Furthermore, the incidence of HF after AMI has not shown a significant decline in a decade. Large cohort studies have showed HF in 13% within 1 month of AMI and in 20%–30% by 1-year post-AMI (Jenča et al., 2021).

The area of AMI is a critical factor affecting the prognosis of AMI and the occurrence of its complications, such as HF, arrhythmias, and cardiogenic shock. Myocardial ischemia‒reperfusion injury (MIRI) may exacerbate existing damage, even accounting for up to approximately 50% of the myocardial infarct area (Hausenloy and Yellon, 2016; Heusch, 2020). Liu et al. (2022) indicates that patients with intramyocardial hemorrhage (IMH) after MIRI have an almost doubled infarct volume during the acute phase of AMI, reaching 85% compared to patients without IMH. A meta-analysis measuring AMI size using cardiovascular magnetic resonance in STEMI patients showed that for every 5% increase in AMI size, the risk of hospitalization due to HF increases by 20% (Stone et al., 2016). On the contrary, Stiermaier et al. (2019) conducted a study on 696 STEMI patients, showing that combined intrahospital remote ischemic preconditioning and postconditioning, compared to patients receiving PCI alone, can reduce the long-term risk of MACE after AMI from 16.9% to 10.2%, and decrease the incidence of HF from 7.8% to 2.7%. Therefore, preventing MIRI is essential for improving the effectiveness of reperfusion therapy to ameliorate AMI. However, there is still a lack of guideline-recommended medicines with significant efficacy for MIRI.

Research has shown that autophagy has a protective effect by inhibiting endoplasmic reticulum (ER) stress, as it relieves ER stress by degrading unfolded or misfolded proteins or the damaged ER to suppress the activation of ER stress-associated apoptosis upon stress injury (Biczo et al., 2018; Guan et al., 2019; Kokten et al., 2018; Li X. et al., 2018). ER-phagy maintains ER homeostasis via the selective degradation of the damaged ER under ER stress conditions. This process is mediated by several receptors on the ER membrane, such as FAM134B, SEC62, RTN3, and CCPG1 (Liang et al., 2019; Zhang et al., 2019). Among these receptors, FAM134B, which is found in many tissues in the human body, is an important contributor to the initial phase of ER stress-induced ER-phagy (Khaminets et al., 2015). FAM134B contains a reticulon homology domain (RHD) that promotes the bending and vesiculation of damaged ER membranes upon phosphorylation-dependent ubiquitination and a cytoplasmic carboxyl-terminal domain (LIR) that interacts with LC3/GABARAP to initiate ER-phagy following further degradation of the damaged ER into small fragments (Berkane et al., 2023; Foronda et al., 2023; González et al., 2023). However, the regulatory role of FAM134B in MIRI is not yet clear.

Qingre Huoxue decoction (QRHX), which is a TCM formula that specifically targets the underlying pathogenesis of “heat poison and blood stasis” syndrome, has been utilized in clinical contexts for more than two decades and has been proven to be clinically effective for treating coronary heart disease (CHD). Previous research has indicated that compared with standard treatment, QRHX has a positive effect on perioperative period within 30 days in patients with AMI, reducing incidence of MACE, including all-cause death and recurrent AMI, as well as the occurrence of MACE after 1 year following PCI, which is an independent protective factor for cardiac death within 1 year in STEMI patients after PCI [HR = 0.271 (0.074, 0.997), *P* = 0.0495] (Zuo et al., 2021). QRHX also reduces myocardial damage in AMI rats by stimulating autophagy via the PI3K/Akt pathway (Jin et al., 2021). In addition, it ameliorates MIRI by decreasing the activation of oxidative stress-related pathways (Li et al., 2022a). However, whether QRHX alleviates MIRI by regulating autophagy and the underlying mechanism remain unclear. In the present study, the ability of QRHX to ameliorate MIRI and the relationship between autophagy and ER stress were further investigated to provide a biological basis for the clinical application of QRHX.

## 2 Materials and methods

### 2.1 Animals and grouping

Adult male SD rats weighing 200 ± 20 g were obtained from the Animal Experimental Center of Southern Medical University (SCXK 2021–0041) in Guangdong, China. The rats were housed in an SPF barrier environment at the First Affiliated Hospital of Guangzhou University of Chinese Medicine (SYXK 2018-0182). The rats were kept at a temperature of 25°C ± 2°C and humidity of 50% ± 5% on a normal 12 h light/dark cycle. All animal experiments were performed following the Guide for the Care and Use of Laboratory Animals and were approved by the Experimental Animal Ethics Committee of the First Affiliated Hospital of Guangzhou University of Chinese Medicine (approval number TCMF1-2021044).

After a 7-day acclimatization period, the rats were assigned to the following 8 groups, with 12 rats in each group, with a random number table: the sham surgery (sham) group; MIRI group; MIRI + low-dose QRHX (QRHX-L) group; MIRI + medium-dose QRHX (QRHX-M) group; MIRI + high-dose QRHX (QRHX-H) group; MIRI + rapamycin (RAPA) group; MIRI + chloroquine (CQ) group; and MIRI + CQ + QRHX-H (QRHX-H + CQ) group. Herbal concentrate granules of QRHX were purchased from Guangdong Efang Pharmaceutical Co., Ltd.; the granules consisted of Scutellaria baicalensis Georgi [Lamiaceae; scutellariae baicalensis radix] (*Huangqin*) (No. 1051771), Ilex pubescens Hook. and Arn. [Aquifoliaceae; ilicis pubescentis radix et cauli](*Maodongqing*) (No. 0117391), Salvia miltiorrhiza Bunge [Lamiaceae; Salviae miltiorrhizae radix et rhizoma] (*Danshen*) (No. 1050741), Paeonia veitchii Lynch [Paeoniaceae; paeoniae rubra radix] (*Chishao*) (No. 1059251), Oreocome striata (DC.) Pimenov and Kljuykov [Apiaceae; ligusticum wallichii] (*Chuanxiong*) (No. 1049241), Dalbergia odorifera T.C.Chen [Fabaceae; dalbergiae odoriferae lignum] (*Jiangxiang*) (No. 1048271), and Carthamus tinctorius L. [Asteraceae; carthami oleum raffinatum] (*Honghua*) (No. 1041191). The low, medium, and high doses of QRHX were 4.94, 9.88, and 19.79 g/kg/d (0.5, 1, and 2 times the standard dose), respectively, and were administered by gavage. The sham group and MIRI group were given an equivalent volume of normal saline intragastrically. RAPA and CQ, used as positive and negative controls for autophagy induction, respectively, were obtained from Shanghai Macklin Biochemical Technology Co., Ltd., and were administered intraperitoneally at dosages of 2.5 mg/kg/d and 20 mg/kg/d, respectively. All the abovementioned treatments were administered for 14 consecutive days before the rats underwent MIRI.

### 2.2 Establishment of the MIRI rat model

The MIRI rat model was created as outlined in a previous study (Li et al., 2022b). Briefly, the rats were anesthetized with 2% pentobarbital sodium (1.5 mL/kg) (No. 57330; Sigma‒Aldrich, United States), intubated with an endotracheal tube and connected to a ventilator. The third rib was cut to expose the heart and tear the pericardium. The LAD artery was positioned 2 mm beneath the middle of the pulmonary conus and the left atrial appendage and was ligated with a 5–0 suture and a 1.0 mm diameter cotton thread. After 30 min of ischemia, the slipknot was loosened to allow reperfusion for 90 min. After the MIRI model was successfully established, blood and heart tissue were subsequently collected from the rats for subsequent experiments. Electrocardiography was performed to assess changes in lead II. Successful modeling was indicated by a change in the color of the left anterior wall myocardium from red to pale and elevation of the ST segment in lead II by more than 0.1 mV. Following reperfusion, the color of the ischemic myocardium became reddish, and the ST segment decreased by more than 50%. The animals in the sham group underwent the same surgical procedures except for LAD occlusion.

### 2.3 Measurement of the myocardial infarct size

After reperfusion, 2 mL of 2% Evans blue (EB) (E6135; Shanghai Macklin Biochemical Technology Co., Ltd.) solution was administered through the celiac vein of the rats after the LAD artery was religated. The heart was removed when the rat’s limbs were dyed blue and then placed at −20°C for 20 min following a rinse with ice-cold saline. The heart tissue below the ligation site was divided into 5 pieces, each 2 mm thick. The sections were subsequently submerged in a 10 mL mixture containing 1% 2,3,5-triphenyltetrazolium chloride (TTC) (T8877, Sigma‒Aldrich) and incubated at 37°C in the dark for 20 min. Afterward, they were fixed with 4% paraformaldehyde (G1101, Wuhan Servicebio Technology Co., Ltd.). Images taken with a digital camera (Nikon SMZ1000, Nikon, Tokyo, Japan) were analyzed via ImageJ software (Bethesda, MD, United States). The white area represented the infarct area (IA), and the nonblue area represented the area at risk (AAR); the infarct size (%) was calculated as IA/AAR, and the ischemic area (%) was calculated as AAR/left ventricular area (LV).

### 2.4 Echocardiography imaging

After reperfusion, LV echocardiography images were collected and analyzed with a VisualSonics Vevo 2100 instrument (Fujifilm, Japan). The probe was placed vertically on the left margin of the mouse parasternal bone, with an azimuth of approximately 20°–30° from the long axis of the mouse body. A long-axis view image of the left ventricle in the parasternal bone was obtained, with the sampling line avoiding the papillary muscle. The left ventricular ejection fraction (EF), left ventricular fractional shortening (FS) and left ventricular end diastolic or systolic diameter (LVID; d or LVID; s) were measured and calculated from M-mode recordings. All measurements were taken from an average of three cardiac cycles.

### 2.5 Analysis of the levels of myocardial enzymes and oxidative stress

After reperfusion, blood samples were collected and centrifuged at 4°C at 3,000 r/min for 15 min. Following serum separation, LDH and CK-MB activities in the serum were measured via an LDH assay kit (S03034, Rayto Life and Analytical Sciences Co., Ltd.) and a CK-MB assay kit (C060, Changchun Huili Biotech Co., Ltd.), respectively, with an automatic biochemical instrument. The serum cTnI concentration was measured via a rat troponin I ELISA kit (MM-0377R1; Jiangsu Meimian Industrial Co., Ltd.) following the manufacturer’s guidelines. The serum levels of superoxide dismutase, malondialdehyde, and glutathione peroxidase were determined with assay kits (A001-1, A003-1, and A005) provided by Nanjing Jiancheng Bioengineering Institute.

### 2.6 Hematoxylin‒eosin (HE) staining

After reperfusion, heart tissue was immersed in 4% paraformaldehyde for 24 h. The sections were subsequently immersed in alcohol, an alcohol-benzene solution and xylene until completely transparent. Paraffin-embedded tissue sections with a thickness of 4 μm were subjected to antigen retrieval by heating, and the tissue membrane was expanded in 45°C water. The slides were placed on polylysine-coated slides, dewaxed with xylene, dehydrated with alcohol, stained with hematoxylin, rinsed with water, and finally stained with eosin for 30 s (G1003, Wuhan Servicebio Technology Co., Ltd.). Afterward, the sections were dried with various concentrations of alcohol, cleared with xylene, and mounted with neutral gum. Pathomorphological changes were observed with a digital pathological section scanner (Pannoramic MIDI Ⅱ, Jinan Danjier Electronics Co., Ltd.).

### 2.7 Terminal deoxynucleotidyl transferase-mediated dUTP nick-end labeling (TUNEL) assay

Heart tissues were rinsed with chilled saline solution, immersed in 4% paraformaldehyde for 24 h, dried, embedded in paraffin, and cut into sections. Cardiomyocytes undergoing apoptosis were detected via a FITC TUNEL cell apoptosis detection kit (G1501, Wuhan Servicebio Technology Co. Ltd.) following the manufacturer’s guidelines. The nuclei were subsequently stained with DAPI. A digital pathological section scanner was used to capture images of the sections. The apoptosis rate was determined via ImageJ software by dividing the number of green fluorescent cells by the number of blue fluorescent cells and multiplying by 100 to obtain the percentage of apoptotic cells.

### 2.8 Preparation of serum from QRHX-treated rats and identification of its main metabolites

Fifty male rats with a body weight of 220 ± 20 g were divided randomly into 2 groups: the QRHX-mediating serum (QRHX-MS) group and the blank serum (BS) group. Rats in the QRHX-MS group received the same dose of QRHX as those in the QRHX-H group via intragastric administration; rats in the BS group were given an equivalent volume of distilled water for 7 consecutive days. Two hours after the final treatment, the rats were anesthetized, and blood samples were obtained from the ventral aorta. After incubation at room temperature for 2 h, the serum was centrifuged at 3,000 rpm for 15 min to separate QRHX-MS or BS. Ultra-performance liquid chromatography‒tandem mass spectrometry (UPLC‒MS) was utilized to identify the primary pharmacological metabolites of QRHX-MS. The original data were processed via Progenesis QI v2.3 software, the baseline data were filtered, aligned and normalized. The chemical metabolites was identified by analyzing their mass numbers, secondary fragments, and isotopic distributions via the HMDB, Lipidmapsv2.3, and METLIN databases for qualitative evaluation. Metabolites with higher levels in QRHX-MS than in BS with screening criteria of *p* < 0.05 and a fold change (FC) of at least 1.2 were identified. The main pharmacological metabolites of QRHX-MS was determined by intersecting the identified ingredients of each herb in QRHX through the TCMSP (https://old.tcmsp-e.com/tcmsp.php), SYMMAP (http://www.symmap.org/) and HERB (http://herb.ac.cn/) databases.

### 2.9 Cell culture and hypoxia/reoxygenation (H/R) exposure

H9c2 cardiomyocytes were cultured in DMEM (PM150210, Procell, China) supplemented with 10% FBS (Gibco) in a 95% air and 5% CO_2_ atmosphere at 37°C. The cells were detached via 0.25% trypsin with EDTA for subculturing until they reached 80% ∼ 90% confluence, after which they were collected for subsequent experiments in the logarithmic growth phase.

After the cells were cultured in glucose-free DMEM in 95% nitrogen and 5% carbon dioxide at 37°C to simulate low oxygen levels, they were then switched to complete DMEM and transferred to an incubator containing 95% air and 5% CO_2_ at 37°C to simulate reoxygenation. The cells were cultured under oxygen deprivation for various durations (3, 6, 12, 18, and 24 h) and under normal oxygen conditions for various durations (0, 3, 6, and 9 h) to establish an H/R model to determine the most effective conditions for modeling.

### 2.10 Treatment with QRHX-MS *in vitro*

To determine the optimal dose and treatment duration of QRHX-MS, H9c2 cells were exposed to various concentrations of QRHX-MS (2.5%, 5%, 10%, and 20%) for several durations (6, 12, 24, 48, and 72 h) prior to H/R. The cells were subsequently assigned to various groups: the blank, control, optimal QRHX-MS concentration, RAPA (5 µM), CQ (1 µM), and CQ + optimal QRHX-MS concentration groups. Additionally, H9c2 cells transfected with FAM134B small interfering RNA (siRNA) or negative control siRNA (siScramble) via Lipo8000 transfection reagent (C0533, Beyotime, China) were divided into 6 groups: the siScramble + blank, siFAM134B+ blank, siScramble + control, siFAM134B + control, siScramble + optimal QRHX-MS concentration or siFAM134B+ optimal QRHX-MS concentration groups. The blank groups were incubated in a 5% CO_2_ environment at 37°C, while the other groups were subjected to H/R following administration of the corresponding treatment.

### 2.11 Cell viability assay

H9c2 cells were seeded in 96-well plates (2 × 10^4^ cells per well) and incubated in a 5% CO_2_ environment at 37°C for 24 h. After the appropriate treatment was administered and the medium of each well was removed, 100 µL of 10% CCK8 solution (CK04-500T; Biosharp, China) was added, and the mixture was incubated at 37°C for 1 h. A microplate reader (3,599, Corning, United States) was used to measure the optical density (OD) at 450 nm.

### 2.12 Flow cytometr

After the appropriate treatment, the cells in each group were digested with EDTA-free pancreatic enzymes and then washed with precooled PBS. Approximately 1×10^6^ cells were aspirated and washed again with 2 mL of 1 × binding buffer. After the cells were resuspended in 1 mL of 1 × binding buffer, 100 µL of the sample was transferred to a flow tube. Following gentle vortexing, Annexin V-FITC (5 µL) and PI (5 µL) (FXP018; 4A Biotech, China) were sequentially added and incubated in the dark at room temperature for 5 min. Finally, 400 µL of PBS was added to the samples, followed by mixing. The percentage of apoptotic cells was measured via flow cytometry (FACSCelesta, BD, United States).

### 2.13 Western blotting

Total protein was extracted from animal tissue or cells via RIPA lysis buffer (P0013; Beyotime, China), and the protein concentration was determined with a BCA assay kit (P0010; Beyotime, China). SDS‒PAGE was used to separate proteins from each group, which were subsequently transferred to a PVDF membrane (P0021S, Beyotime, China) with western rapid transfer buffer (P0572, Beyotime, China). Next, the membrane was treated with blocking solution (P0252, Beyotime, China) at ambient temperature for 1 h. Afterward, the membrane was incubated with primary antibodies against GRP78 (66,574; Proteintech), CHOP (L63F7; CST), Bax (60,267; Proteintech), Bcl-2 (60,178; Proteintech), LC3B (E5Q2K; CST), P62 (66,184; Proteintech), FAM134B (E8Y9R; CST), or β-actin (66,009; Proteintech) overnight at 4°C. The membrane was subsequently washed three times with western wash buffer (P0023C6; Beyotime, China) before being incubated with the appropriate secondary antibodies at room temperature for 1 h. After three more washes, the membrane was immersed in an ultrasensitive chemiluminescent substrate (P0018AM; Beyotime, China), and the protein bands were visualized via a chemiluminescence device (Bio-Rad, United States). The gray values of the bands were measured via ImageJ software.

### 2.14 qPCR

Processed tissues or cells were collected and washed with saline or PBS. RNA was extracted with an RNA purification kit (B0004D, EZBioscience) and then reverse transcribed into cDNA, which was amplified via a Color Reverse Transcription Kit (A0010CGQ, EZBioscience). cDNA from each group was amplified with a Color SYBR Green qPCR Master Kit (A0012-R2, EZBioscience) on a CFX Connect Real-Time PCR System (Bio-Rad). For amplification, the procedure began with a 30 s preheating step at 95°C, followed by 35 cycles of denaturation at the same temperature for 5 s, annealing at 56°C–60°C for 30 s, and extension at 72°C for 10 min. The 2^−ΔΔCT^ method was used to compare the expression levels of target genes among various groups (Table 1).

**TABLE 1:** Sequences of primers for gene amplification.

Gene name	Forward	Reverse
*GRP78*	CTT​CTA​GGC​ATC​CCT​TCC​TTA​CAG​C	GAA​GCG​CTC​ACG​AGA​CAG​GTG​GA
*CHOP*	GGA​GGT​CCT​GTC​CTC​AGA​TGA​A	GCT​CCT​CTG​TCA​GCC​AAG​CTA​G
*Caspase3*	GGA​GCT​TGG​AAC​GCG​AAG​AA	ACA​CAA​GCC​CAT​TTC​AGG​GT
*BAX*	ACA​CCT​GAG​CTG​ACC​TTG​GA	AGT​TCA​TCG​CCA​ATT​CGC​CT
*Bcl-2*	CTG​GTG​GAC​AAC​ATC​GCT​CT	GCA​TGC​TGG​GGC​CAT​ATA​GT
*LC3B*	AGC​TCT​GAA​GGC​AAC​AGC​AAC​A	GCT​CCA​TGC​AGG​TAG​CAG​GAA
*Beclin-1*	GAA​ACT​GGA​CAC​GAG​CTT​CAA​GA	ACC​ATC​CTG​GCG​AGT​TTC​AAT​A
*P62*	AAG​CTG​CCC​TGT​ACC​CAC​ATC	ACC​CAT​GGA​CAG​CAT​CTG​AGA​G
*FAM134B*	ACC​CAC​AGA​GCT​CAA​GAC​AA	CTG​GTC​TTT​GAT​GGC​AGC​TG
*PERK*	CTT​TCG​GTG​CTC​CAA​GGC​TC	TTA​CTA​AGG​ACC​TGC​CGC​GA
*β-actin*	GGA​GAT​TAC​TGC​CCT​GGC​TCC​TA	GAC​TCA​TCG​TAC​TCC​TGC​TTG​CTG

### 2.15 Immunofluorescence colocalization analysis

Left ventricular myocardial tissue was isolated, treated with paraformaldehyde, dehydrated, embedded in paraffin, sectioned consecutively, deparaffinized using xylene, and finally rehydrated with a graded series of ethanol solutions. The sections were subjected to antigen retrieval by soaking them in a heated solution for antigen repair at 95°C and then blocked with 10% goat serum on slides for 1 h. Afterward, the samples were incubated overnight at 4°C with 100 μL of primary antibodies against LAMP2 (66,301, Proteintech), FAM34B, and LC3B in a humid chamber. After washing three times, 100 μL of appropriate diluted fluorescent secondary antibodies was added, followed by incubation at 37°C for 1 h. Subsequently, counterstaining with DAPI was performed for 5 min in the dark. The slices were sealed with mounting medium containing an anti-fluorescence quencher and scanned with a digital pathological slide scanner. LC3 appeared as bright green dots, LAMP2 or FAM134B appeared as bright red dots, and autolysosomes or ER-phagosomes appeared as yellow dots. The mean fluorescence intensity in each group was analyzed with ImageJ.

### 2.16 Transmission electron microscopy (TEM)

After rapid isolation, fresh heart tissues (1 × 1 × 1 mm^3^) were immersed in 1% osmium tetroxide solution for 1 h after fixation with 2.5% glutaraldehyde overnight at 4°C. The samples were subsequently dehydrated in ethanol and embedded in Epon 812. The permeabilized samples were embedded in epoxy resin and cut into 100-nm-thick slices by using an ultramicrotome (Leica UC7, DE), followed by observation of double-membrane autophagosomes encapsulating the ER via transmission electron microscopy (HITACHI, HT7800, Japan).

### 2.17 Evaluation of autophagic flux

Fresh culture medium containing the virus AdPlus-mCherry-GFP-LC3 (C3012; Beyotime, China) at a multiplicity of infection (MOI) of 40 was added to the cells, which were subsequently incubated for 24 h. Afterward, the culture medium containing the virus was removed, and the cells were subjected to the appropriate pretreatment. Then, 10 mL of Hoechst 33,342 staining solution (C1027; Beyotime, China) was added to each well, followed by incubation at 37°C in the dark for 10 min. Later, a confocal laser scanning microscope (Olympus, Tokyo, Japan) was used for observation. GFP is a green fluorescent protein that is easily quenched in the acidic environment of lysosomes, and mCherry is a stable red fluorescent protein. Therefore, yellow dots represented autophagosomes, and red fluorescent dots represented autolysosomes. Fluorescent mCherry and GFP puncta were counted via ImageJ.

### 2.18 siRNA transfection

Lipo8000 transfection reagent was used to transfect H9c2 cells with siScramble or FAM134B siRNA. Total RNA or protein was extracted from the cells 48 h post-transfection for qPCR or Western blotting to assess the transfection efficiency. The siRNA sequence with the best knockdown efficiency was selected for subsequent experiments. Shanghai Sangon Biotech created and produced the siRNAs (Table 2).

**TABLE 2:** Sequences of the FAM134B siRNAs.

siRNA name	Sense (5’-3’)	Antisense (5’-3’)
*FAM134B-1145*	CCA​CAG​AGC​UCA​AGA​CAA​ATT	UUU​GUC​UUG​AGC​UCU​GUG​GTT
*FAM134B-550*	GCA​GAA​UCA​UGG​AUG​AAU​UTT	AAU​UCA​UCC​AUG​AUU​CUG​CTT
*FAM134B-1446*	GGG​ACU​AAC​ACA​AGA​CCA​ATT	UUG​GUC​UUG​UGU​UAG​UCC​CTT
*Negative control*	UUC​UCC​GAA​CGU​GUC​ACG​UTT	ACG​UGA​CAC​GUU​CGG​AGA​ATT

### 2.19 Statistical analysis

The data from the above experiments were analyzed with SPSS 24.0 (IBM, Armonk, NY, United States). Normally distributed quantitative data are presented as the means ± standard deviations. ANOVA was used to compare normally distributed data among multiple groups. If the assumption of homogeneity of variance was met, the least significant difference (LSD) test was applied for further comparisons. However, if the assumption of variance was not satisfied, Dunnett’s T3 test was used. A *p*-value less than 0.05 indicated statistical significance. Statistical graphs were generated with GraphPad Prism 8.0 software (GraphPad Software, United States).

## 3 Results

### 3.1 QRHX decreased the myocardial infarct size, the levels of cardiac enzymes and oxidative stress after MIRI

**FIGURE 1:**
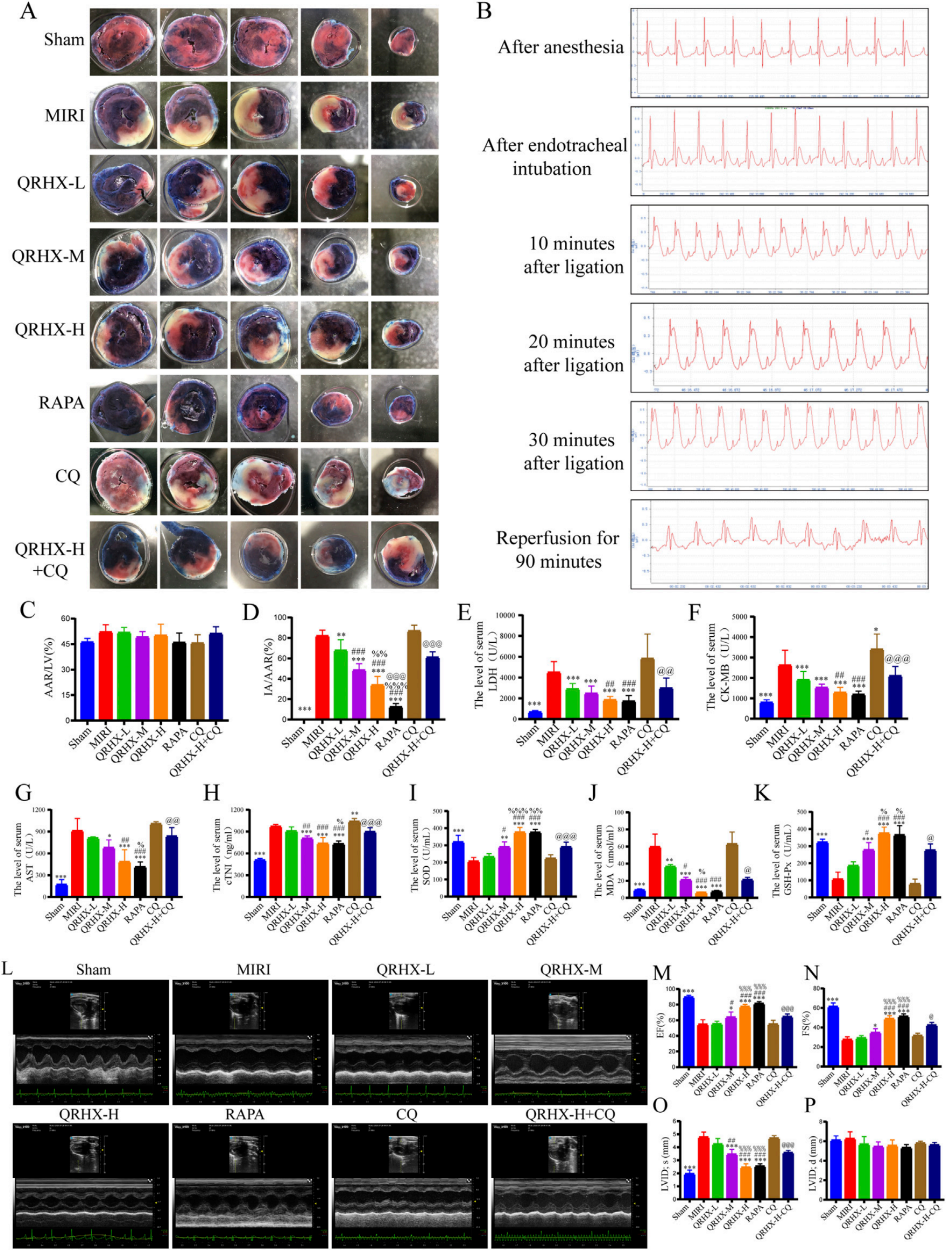
QRHX attenuates the myocardial infarct size, serum myocardial enzyme levels, oxidative stress severity and improves cardiac function in MIRI rats. **(A)** After 90 min of reperfusion, the left ventricles of the rats in each group were collected and stained with EB/TTC to evaluate the extent of myocardial infarction and the AAR of myocardial ischemia. **(B)** ECG of a rat recorded during the MIRI modeling process. **(C)** The ratio of the AAR of ischemia to the total ventricular area was calculated for each group (*n* = 6). **(D)** The ratio of the myocardial infarct area to the AAR of ischemia was calculated for each group (*n* = 6). **(E–H)** Myocardial levels of enzymes, including LDH, CK-MB, AST, and cTNI, in each group (*n* = 6). **(J–K)** Serum levels of oxidative stress indicators, including SOD, MDA, and GSH-Px, in each group (*n* = 6). **(L)** M-model echocardiography of rats. **(M–P)** Comparison of the EF, FS, LVID; s, LVID; d among groups (*n* = 6). Significance levels are denoted as follows: ^*^ *p* < 0.05, ^**^ *p* < 0.01, ^***^ *p* < 0.001 in comparison to the MIRI group; ^#^ *p* < 0.05, ^##^ *p* < 0.01, ^###^ *p* < 0.001 in comparison to the QRHX-L group; ^%^ *p* < 0.05, ^%%^ *p* < 0.01, ^%%%^ *p* < 0.001 in comparison to the QRHX-M group; and ^@^ *p* < 0.05, ^@@^ *p* < 0.01, ^@@@^ *p* < 0.001 in comparison to the QRHX-H group. All the data are presented as the means ± SDs.

After 30 min of ischemia induced by ligation of the rat LAD artery and 90 min of reperfusion following the removal of the ligature, the ST segment decreased by more than 50%, indicating successful establishment of the MIRI rat model (Figure 1B). There were no significant differences observed in the nonblue area among the experimental groups, indicating a consistent degree of ischemic damage after ligation (Figures 1A,C). Compared with that in the sham group, the myocardial infarct area in the MIRI group was significantly larger (*p* < 0.01). Compared with that in the MIRI group, the infarct area in the QRHX groups decreased in a dose-dependent manner (*p* < 0.01). Furthermore, the infarct area in the RAPA group was smaller than that in the QRHX-H group (*p* < 0.05), whereas CQ partially inhibited the cardioprotective effect of QRHX (*p* < 0.05) (Figures 1A, D).

Elevated levels of LDH, CK-MB, AST, cTNI and MDA and reductions in SOD and GSH-Px levels were observed in the MIRI group compared to the sham group (*p* < 0.01). LDH, CK-MB, AST, cTNI and MDA levels decreased (*p* < 0.001), whereas SOD and GSH-Px levels increased, in the QRHX-treated groups in a dose-dependent manner (*p* < 0.001). There were no significant differences in the levels of cardiac enzymes or oxidative stress between the RAPA group and the QRHX-H group. However, CQ partially reversed the protective effect of QRHX (*p* < 0.01) (Figures 1E–K).

These results indicate that QRHX alleviates myocardial infarction, cardiac injury and oxidative stress caused by MIRI.

### 3.2 QRHX improves cardiac function and morphology in MIRI

QRHX also exerts protective effects on cardiac function and morphology in MIRI rats. As illustrated in Figures 1L–P, the EF was significantly reduced from 89.28% in the MIRI group to 54.42% in the sham group (*p* < 0.001), and FS was significantly reduced from 61.15% to 27.54% (*p* < 0.001); however, LVID; s was significantly elevated by 1.44 times in the MIRI group compared to the sham group (*p* < 0.001). QRHX improved the aforementioned cardiac function indicators in a dose-dependent manner (*p* < 0.05), exerting a similar effect as RAPA, while CQ partially counteracted the beneficial effects of QRHX (*p* < 0.05).

**FIGURE 2:**
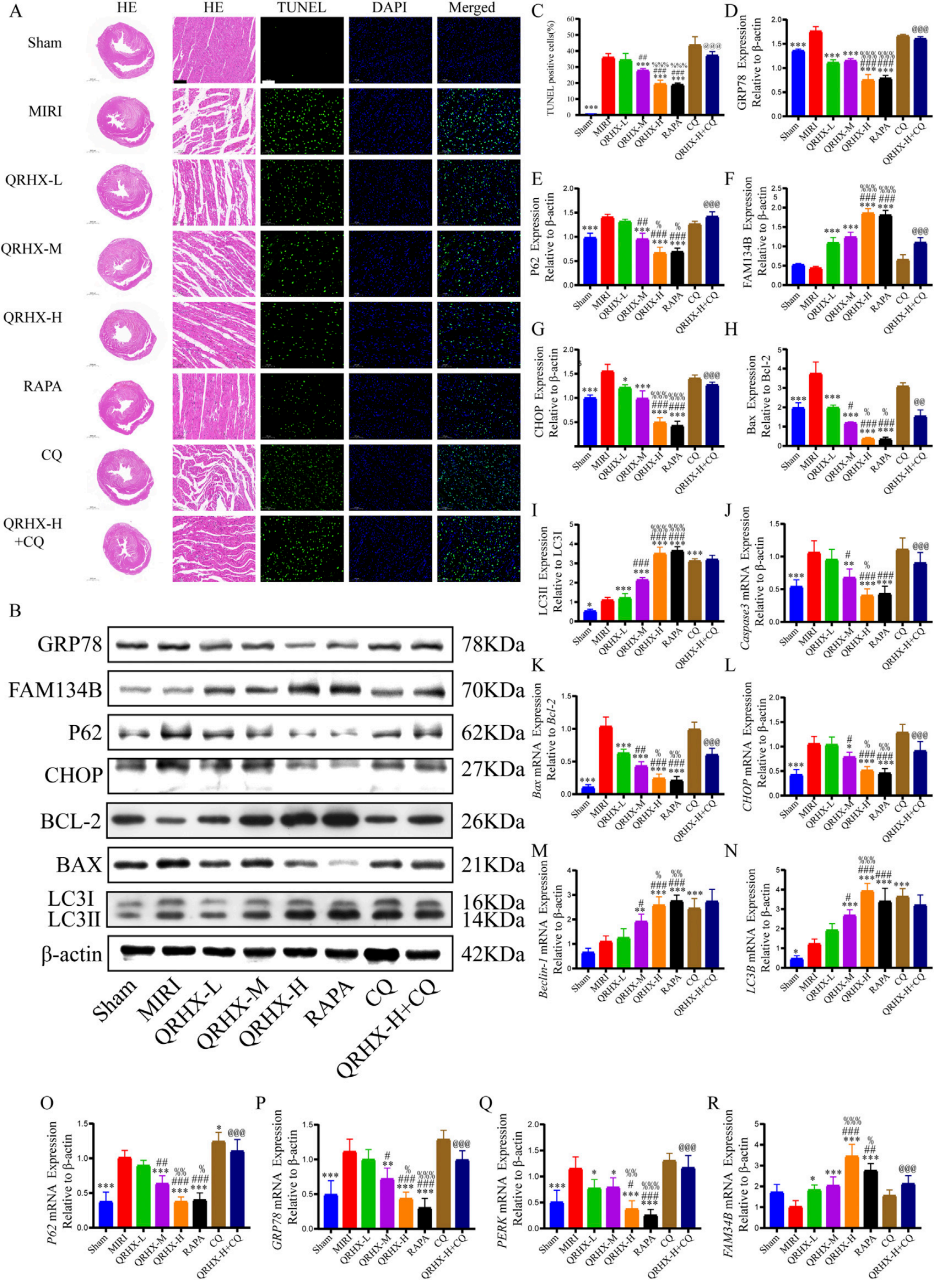
QRHX promotes autophagy in the myocardium to inhibit ER stress and myocardial tissue damage and apoptosis after MIRI. **(A)** Ventricular slices collected from each group at the end of ischemia‒reperfusion was subjected to HE or TUNEL staining (200×); scale bar = 100 μm. **(B)** The protein levels of autophagy-, ER stress-, and apoptosis-related proteins in heart tissues from all groups were analyzed using Western blotting. **(C)** Apoptosis rates of the myocardial cells in each group (*n* = 6). **(D–I)** Western blotting was performed to analyze the expression of GRP78, P62, FAM134B, and CHOP, the Bax/Bcl-2 ratio, and the LC3II/LC3I ratio *in vivo* (*n* = 3). **(J‒R)** qPCR was used to compare the mRNA expression levels of Caspase-3, CHOP, Beclin-1, LC3B, P62, GRP78, PERK, and FAM134B and the Bax/Bcl-2 ratio among the various groups (*n* = 6). Significance levels are denoted as follows: ^*^ *p* < 0.05, ^**^ *p* < 0.01, ^***^ *p* < 0.001 in comparison to the MIRI group; ^#^ *p* < 0.05, ^##^ *p* < 0.01, ^###^ *p* < 0.001 in comparison to the QRHX-L group; ^%^ *p* < 0.05, ^%%^ *p* < 0.01, ^%%%^ *p* < 0.001 in comparison to the QRHX-M group; and ^@^ *p* < 0.05, ^@@^ *p* < 0.01, ^@@@^ *p* < 0.001 in comparison to the QRHX-H group. All the data are presented as the means ± SDs.

Compared with the sham group, the MIRI group exhibited more severe pathological damage, including myocardial swelling and deformation, myofibril rupture, interstitial edema, and leukocyte infiltration. QRHX ameliorated pathological damage to a greater degree with increasing dose. In the RAPA group, the myocardium was relatively orderly arranged, the cardiomyocyte swelling and interstitial edema resembling those in the QRHX-H group. CQ partially reversed the protective effect of QRHX (Figure 2A).

Moreover, pretreatment with QRHX reduced cardiomyocyte apoptosis in MIRI rats. The percentage of apoptotic cells in the MIRI group was markedly greater than that in the sham group (*p* < 0.001). Compared with the MIRI group, the QRHX-M and QRHX-H groups exhibited substantial dose-dependent reductions in apoptosis (*p* < 0.001). There was no notable difference in apoptosis between the RAPA and QRHX-H groups. However, CQ partially inhibited the ability of QRHX to decrease apoptosis (*p* < 0.001) (Figures 2A, C). These findings suggest that QRHX improves cardiac function and reduces pathomorphological changes and apoptosis in the myocardium during MIRI.

### 3.3 QRHX inhibits ER stress and apoptosis by promoting ER-phagy in MIRI

Next, we investigated the impact of QRHX and its relationship with the autophagy‒ER stress axis in MIRI rats. The Bax/Bcl-2 ratio and the protein levels of GRP78, CHOP, and P62 were significantly greater in the MIRI group than in the sham group (*p* < 0.01). QRHX dose-dependently decreased the levels of these proteins but also increased the LC3II/LC3I ratio and the protein level of FAM134B (*p* < 0.01). Additionally, there was no notable variation in the expression levels of these proteins between the RAPA and QRHX-H groups. CQ partially inhibited the ability of QRHX to decrease the Bax/Bcl-2 ratio and GRP78, CHOP, and P62 levels and increase FAM134B levels (*p* < 0.05) but did not affect the LC3II/LC3I ratio (Figures 2B, D–I).

Gene expression analysis yielded similar results. Compared with those in the sham group, the *Bax/Bcl-2* ratio and the gene expression levels of caspase3, *GRP78, CHOP, P62*, and *PERK* in the MIRI group were significantly greater (*p* < 0.01). Compared with the MIRI group, the QRHX groups presented a dose-dependent significant decrease in the expression of these genes and an increase in the expression of *LC3B, FAM134B*, and *Beclin-1* (*p* < 0.01). There was no notable variation in gene expression between the RAPA and QRHX-H groups. CQ partially inhibited the effects of QRHX on the mRNA expression of all of the abovementioned genes except for *LC3B* and *Beclin-1* (Figures 2J–R).

**FIGURE 3:**
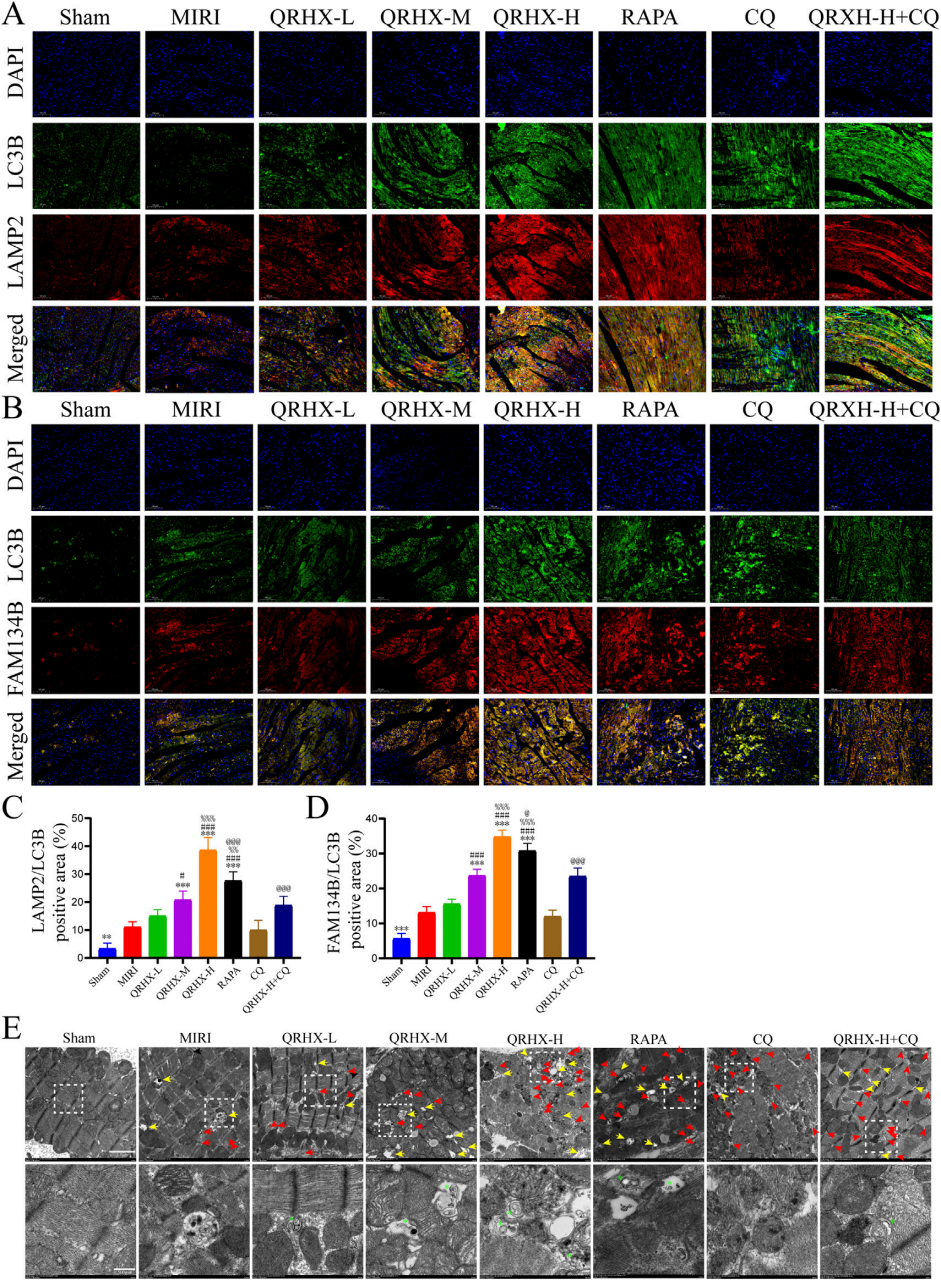
QRHX promotes the fusion of autophagosomes and lysosomes while activating ER-phagy mediated by FAM134B in the myocardium after MIRI. **(A)** Immunofluorescence was used for colocalization analysis of LC3B and LAMP2 in myocardial tissue slices from all groups. **(B)** Immunofluorescence colocalization analysis of LC3B and FAM134B in myocardial tissue from each group. **(C)** Comparison of the degree of LC3B and LAMP2 colocalization in myocardial tissue sections from each group (*n* = 6). **(D)** Comparison of the degree of LC3B and FAM134B colocalization in myocardial tissue sections from each treatment group (*n* = 6). **(E)** TEM images of cardiomyocytes in each group. The yellow arrows point to autolysosomes. The red arrows point to autophagosomes. The green asterisks indicate ER fragments inside autophagosomes or autolysosomes. The lower panels show enlarged insets. Scale bar: 2 μm or 500 nm. Significance levels are denoted as follows: ^*^ *p* < 0.05, ^**^ *p* < 0.01, ^***^ *p* < 0.001 in comparison to the MIRI group; ^#^ *p* < 0.05, ^##^ *p* < 0.01, ^###^ *p* < 0.001 in comparison to the QRHX-L group; ^%^ *p* < 0.05, ^%%^ *p* < 0.01, ^%%^ *p* < 0.001 in comparison to the QRHX-M group; and ^@^ *p* < 0.05, ^@@^ *p* < 0.01, ^@@@^ *p* < 0.001 in comparison to the QRHX-H group. All the data are presented as the means ± SDs.

We further explored the relationship between the effects of QRHX and ER-autophagic flux. The average fluorescence intensity of merged LAMP2/LC3B and FAM134B/LC3B signals were notably greater in the MIRI group than in the sham group (*p* < 0.01). Compared with those in the MIRI group, the fluorescence intensity of merged LAMP2/LC3B and FAM134B/LC3B signals in the QRHX groups increased in a dose-dependent manner (*p* < 0.05). Compared with that in the QRHX-H group, the colocalization of LAMP2 and LC3B and of FAM134B and LC3B in the RAPA group was decreased (*p* < 0.001), whereas CQ partially suppressed the ability of QRHX to increase the colocalization of LAMP2 and LC3B and of FAM134B and LC3B (*p* < 0.001) (Figures 3A–D).

TEM analysis was then conducted to observe autophagy-related changes in cardiomyocytes in each group. As shown in Figure 3E, marked induction of cardiomyocyte autophagy was not observed in the sham group, whereas some autophagosomes and autolysosomes were present in the MIRI group. Additionally, QRHX accelerated the formation of ER-phagosomes, autophagosomes containing ER whorls, and autolysosomes in a dose-dependent manner, exerting effects similar to those of RAPA. However, fewer ER-phagosomes and autolysosomes were observed in the QRHX-H + CQ group than in the QRHX-H group.

These findings suggest that QRHX alleviates MIRI-induced ER stress and apoptosis by facilitating autophagic flux, which is associated with FAM134B-mediated ER-phagy.

### 3.4 Analysis of the main pharmacological metabolites of QRHX-MS

**FIGURE 4:**
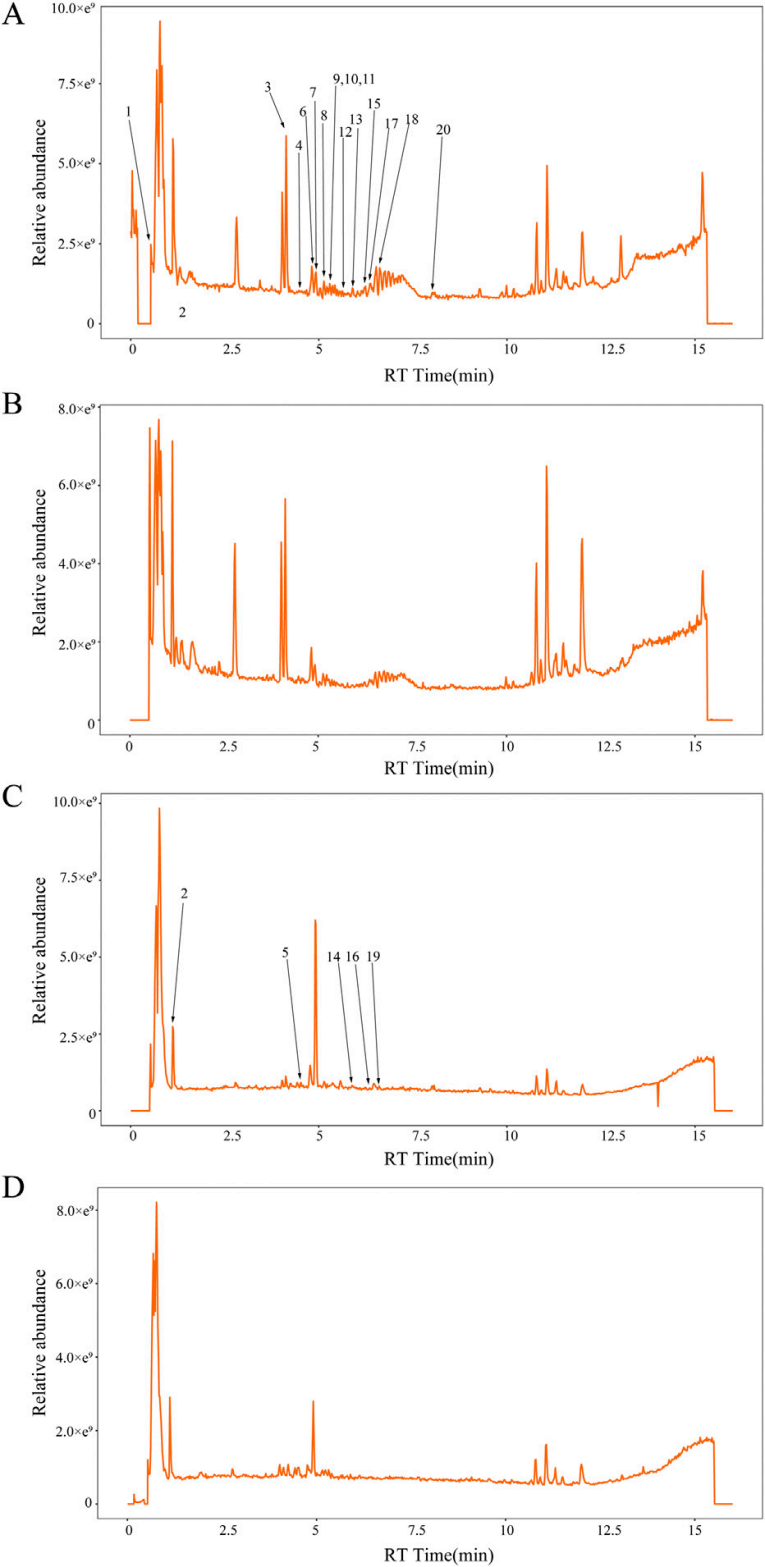
The concentrations of twenty specific chemical metabolites were found to be greater in QRHX-MS than in BS via UPLC‒MS. **(A)** Positive and **(C)** negative ion maps for QRHX-MS. **(B)** Positive and **(D)** negative ion maps for BS. The numbers in the figure correspond to the peak numbers in Table 3. (This part of the content has not been changed).

UPLC‒MS revealed a total of 1,137 chemical metabolites with relatively greater levels in QRHX-MS than in BS. After these metabolites were intersected with the 1,402 chemical ingredients of Scutellaria baicalensis Georgi (*Huangqin*)*,* Ilex pubescens Hook. and Arn (*Maodongqing*)*,* Oreocome striata (DC.) Pimenov and Kljuykov (*Chuanxiong*)*,* Paeonia veitchii Lynch (*Chishao*)*,* Dalbergia odorifera T.C.Chen (*Jiangxiang*)*,* Carthamus tinctorius L. (*Honghua*)*,* and Salvia miltiorrhiza Bunge (*Danshen*) via TCM databases, 20 main active metabolites of QRHX-MS, including baicalin, succinic acid, baicalein, cryptotanshinone, isoferulic acid, 3,4-dihydroxybenzaldehyde, salvianolic acid G, caffeic acid, and isocarthamidin, were identified. Among these metabolites, baicalin had the highest relative content (Figures 4A–D; Table 3).

**TABLE 3:** Through UPLC‒MS, 20 chemical compounds in QRHX‒MS but not in BS were identified.

No.	Peak no.	RT (min)	Ion mode	Formula	Metabolites	Stem from	Compound ID	Intensity
1	13	5.873	pos	C_21_H_18_O_11_	Baicalin	*Chishao, Honghua, Danshen, Huangqin*	49,507	3,768,319.50
2	12	5.596	pos	C_16_H_12_O_5_	Oroxylin A	*Huangqin*	49,521	94,643.56
3	2	1.182	neg	C_4_H_6_O_4_	Succinic acid	*Honghua, Danshen*	HMDB0000254	65,644.84
4	7	4.771	pos	C_7_H_6_O_2_	Benzoic acid	*Chuanxiong, Chishao, Honghua, Danshen, Maodongqing, Huangqin*	HMDB0001870	61,175.21
5	15	6.203	pos	C_16_H_12_O_5_	Wogonin	*Huangqin*	49,754	57,291.38
6	14	5.881	neg	C_15_H_10_O_5_	Baicalein	*Chishao, Honghua, Danshen, Huangqin*	LMPK12111095	38,237.55
7	20	7.835	pos	C_19_H_20_O_3_	Cryptotanshinone	*Danshen*	90,535	25,976.31
8	11	5.421	pos	C_10_H_10_O_4_	Vanillin acetate	*Chuanxiong*	HMDB0029663	23,415.60
9	6	4.693	pos	C_10_H_10_O_4_	Isoferulic acid	*Danshen*	HMDB0000955	20,130.01
10	5	4.642	neg	C_12_H_18_N_2_O_12_	Cibarian	*Chuanxiong*	67,004	17,428.96
11	19	6.606	neg	C_16_H_14_O_6_	5,7,4’- trihydroxy-8-methoxyflavanone	*Huangqin*	LMPK12140670	11,806.56
12	3	4.177	pos	C_7_H_6_O_3_	3,4-Dihydroxybenzaldehyde	*Danshen*	HMDB0059965	10,040.93
13	10	5.342	pos	C_20_H_18_O_10_	Salvianolic acid G	*Danshen*	HMDB0041774	7,985.82
14	1	0.557	pos	C_7_H_8_O_2_	Guaiacol	*Chuanxiong, Chishao, Jiangxiang*	HMDB0001398	6,370.98
15	4	4.463	pos	C_9_H_8_O_4_	Caffeic Acid	*Chuanxiong, Honghua, Danshen*	3,316	5,887.34
16	9	5.322	pos	C_15_H_12_O_6_	Isocarthamidin	*Honghua, Huangqin*	LMPK12140667	4,522.90
17	16	6.233	neg	C_16_H_12_O_5_	Acacetin	*Huangqin*	48,899	3,485.53
18	18	6.471	pos	C_12_H_12_O_2_	Butylidenephthalide	*Chuanxiong*	71,436	3,224.82
19	8	5.148	pos	C_16_H_14_O_6_	7,2’,6’-Trihydroxy-5-methoxyflavanone	*Huangqin*	LMPK12140122	1,528.34
20	17	6.279	pos	C_14_H_12_O_4_	Cearoin	*Jiangxiang*	44,636	1,056.62

RT, Retention time. The compounds listed in the table are sorted according to their relative contents.

### 3.5 QRHX-MS alleviates H/R injury in H9c2 cardiomyocytes

**FIGURE 5:**
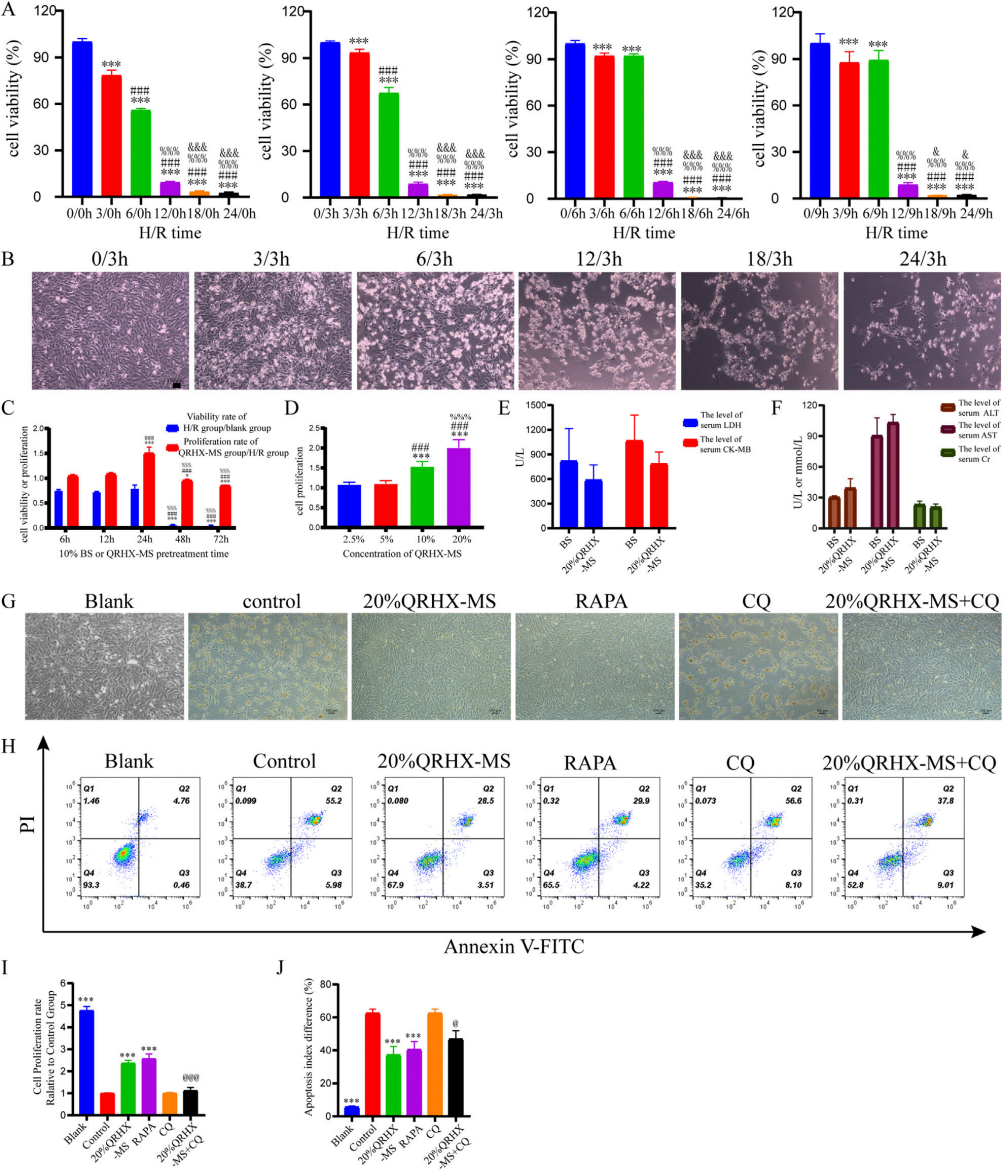
QRHX-MS decreases H/R-induced damage and apoptosis in H9c2 cells. **(A)** H9c2 cell proliferation was evaluated via the CCK-8 assay after H/R for different durations (*n* = 9). ^***^ *p* < 0.001 compared with the hypoxia group at 0 h; ^###^ *p* < 0.001 compared with the hypoxia group at 3 h; ^%%^ *p* < 0.001 compared with the hypoxia group at 6 h; and ^&^ *p* < 0.05 and ^&&&^ *p* < 0.001 compared with the hypoxia group at 12 h. **(B)** Following a 3 h reoxygenation period after hypoxia for different durations, changes in the morphology of H9c2 cells were observed (×100), scale bar = 100 μm. **(C)** The viability and proliferation rates of H/R-exposed H9c2 cells were evaluated after treatment with 10% QRHX-MS or BS for various durations via the CCK-8 assay (*n* = 6); ^*^ *p* < 0.05 and ^***^ *p* < 0.001 compared with the 6 h group; ^###^ *p* < 0.001 compared with the 12 h group; and ^%%%^ *p* < 0.001 compared with the 24 h group. **(D)** The growth rate of H/R-exposed H9c2 cells following treatment with various doses of QRHX-MS was determined via the CCK-8 assay (*n* = 9); ^***^ *p* < 0.001 in comparison to the 2.5% group; ^###^ *p* < 0.001 in comparison to the 5% group; and ^%%%^ *p* < 0.001 in comparison to the 10% group. **(E–F)** Comparison of the safety of QRHX-MS and BS *in vivo* (*n* = 3). **(G)** The appearance of H9c2 cells in different groups after H/R with various pretreatments (100×). **(H, J)** Flow cytometry was used to assess the rate of H9c2 cell apoptosis following H/R in different pretreatment groups (*n* = 4). **(I)** H9c2 cell viability following H/R was assessed in different pretreatment groups via the CCK-8 assay (*n* = 6). ^***^ *p* < 0.001 compared with the control group; ^@^ *p* < 0.05 and ^@@@^ *p* < 0.001 compared with the 20% QRHX-MS group. All the data are presented as the means ± SDs.

To confirm the protective effect of QRHX *in vitro*, experiments were performed on H9c2 cardiomyocytes exposed to H/R. Initially, we evaluated the optimal H/R duration for constructing the H/R model, along with the optimal concentration and most effective treatment duration for QRHX-MS. The results indicated that after the cells were exposed to 6 h of hypoxia followed by 3 h of reoxygenation, the average survival rate was 67.41% (*p* < 0.001). On the basis of the IC50 value, we selected 6 h of hypoxia and 3 h of reoxygenation as the parameters for modeling H/R in subsequent experiments (Figures 5A, B).

Pretreating the cells with 10% serum for 6, 12, or 24 h prior to H/R increased their survival rates to over 50%. Compared with that of cells pretreated with 10% QRHX-MS for 6 h or 12 h, the proliferation rate of cells pretreated with 10% QRHX-MS for 24 h before H/R was greater (*p* < 0.001). Furthermore, compared with that of cells treated with 10% or 5% QRHX-MS, the proliferation rate of cells pretreated with 20% QRHX-MS for 24 h was greater (*p* < 0.001) (Figures 5C, D). Therefore, pretreatment with 20% QRHX-MS for 24 h was subsequently used as the pretreatment regimen. Moreover, there were no notable differences in the levels of safety-related indices, including cardiac enzyme levels, liver enzyme levels and kidney function, between the blank group and the 20% QRHX-MS group, suggesting that QRHX-MS did not have adverse effects on the rats (Figures 5E, F).

Compared with the control group, both the 20% QRHX-MS group and the RAPA group presented a significant increase in proliferation (*p* < 0.001). Compared with that in the 20% QRHX-MS group, the growth rate in the 20% QRHX-MS + CQ group was significantly lower (*p* < 0.001) (Figures 5G, I). The percentage of apoptotic cells in the 20% QRHX-MS and RAPA groups was lower than that in the control group (*p* < 0.001), and the percentage of apoptotic cells was significantly greater in the 20% QRHX-MS + CQ group than in the 20% QRHX-MS group (*p* < 0.001) (Figures 5H, J). The results revealed that QRHX-MS suppressed H/R-induced cardiomyocyte damage and apoptosis.

### 3.6 QRHX-MS increases autophagic flux to prevent ER stress and CHOP-mediated apoptosis in H/R-exposed cardiomyocytes

**FIGURE 6:**
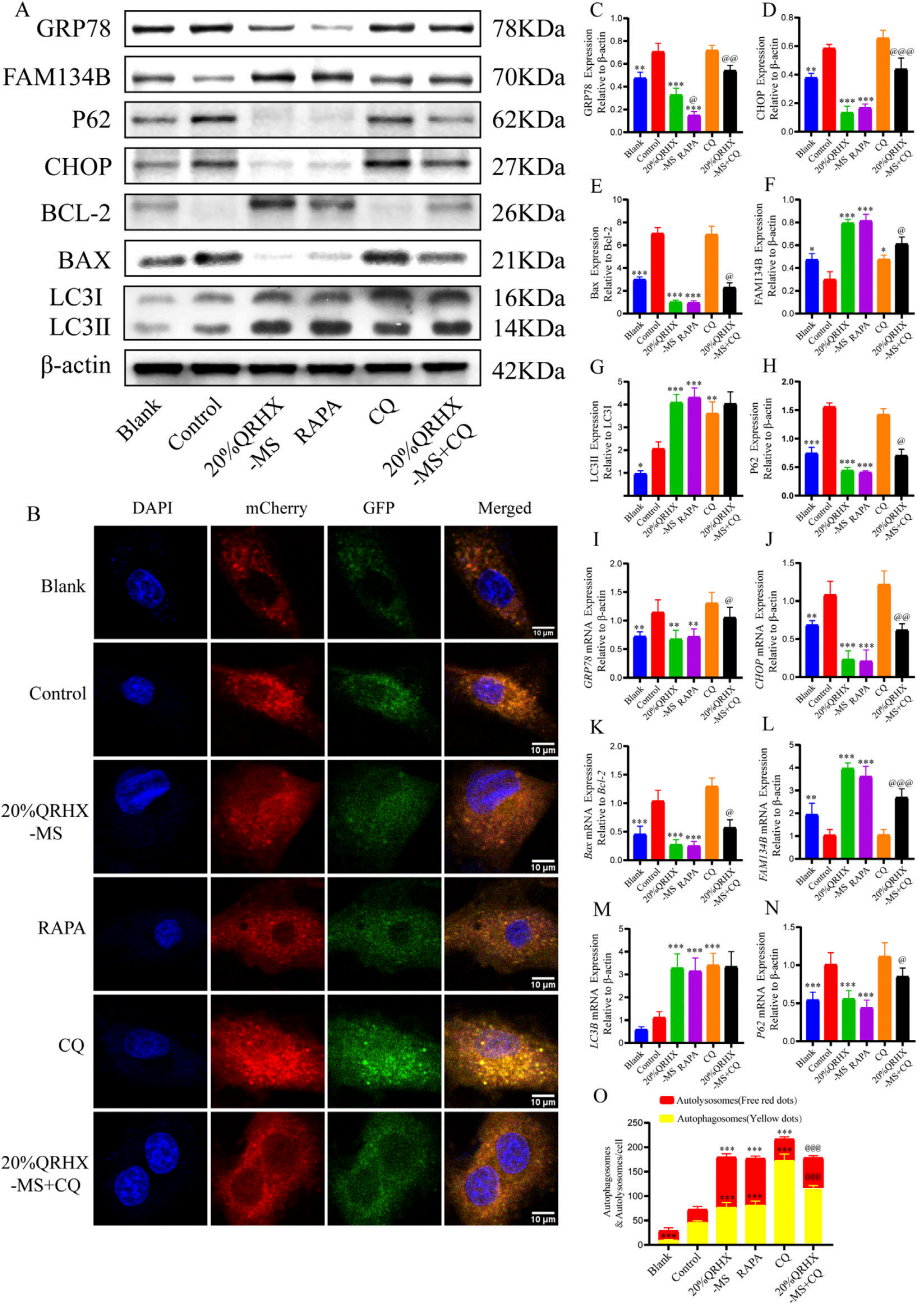
QRHX-MS increases autophagic flux in H9c2 cells exposed to H/R to prevent ER stress and consequent apoptosis. **(A)** The expression of proteins associated with autophagy, ER stress, and apoptosis in each of the pretreatment groups was analyzed via Western blotting. **(B, O)** Images depicting mCherry-GFP-LC3 puncta in H/R-exposed cardiomyocytes from different pretreatment groups, along with the number of autophagosomes (yellow dots) and autolysosomes (red dots) per cell. The significance levels on the yellow and red lines indicate the significance of difference between the respective autophagosome or autolysosome groups (*n* = 6). Scale bar, 10 μm. **(C–H)** In vitro, the expression of GRP78, CHOP, FAM134B, and P62 and the Bax/Bcl-2 and LC3II/LC3I ratios were quantified (*n* = 3). **(I–N)** Comparisons of the mRNA expression of GRP78, CHOP, FAM134B, LC3B and P62 and the Bax/Bcl-2 ratio among the different groups were conducted via qPCR (*n* = 6). ^*^ *p* <0.05, ^**^ *p* <0.01, and ^***^ *p* <0.001 compared with the control group and ^@^ *p* < 0.05, ^@@^p < 0.01, and ^@@@^ *p* < 0.001 compared with the 20% QRHX-MS group. All the data are presented as the means ± SDs.

Compared with those in the blank group, the protein levels of Bax/Bcl-2, P62, GRP78, CHOP, and LC3II/LC3I (*p* < 0.01) in the control group were significantly greater, and FAM134B expression was significantly lower (*p* < 0.05). Compared with the control group, the 20% QRHX-MS group presented a greater LC3II/LC3I ratio and higher protein expression of FAM134B (*p* < 0.001), along with a lower Bax/Bcl-2 ratio and lower P62, CHOP, and GRP78 expression (*p* < 0.01). Furthermore, there were no notable differences in protein expression between the 20% QRHX-MS and RAPA groups. The impact of 20% QRHX-MS on protein expression but not the LC3II/LC3I ratio was counteracted by treatment with CQ (*p* < 0.05) (Figures 6A, C–H). The corresponding gene expression patterns were largely consistent with the abovementioned protein expression patterns (Figures 6I–N).

After H9c2 cells were transfected with the virus AdPlus-mCherry-GFP-LC3B, the number of autophagosomes in the control group was significantly greater than that in the blank group, with no significant difference in the number of autolysosomes. Compared with the control group, the 20% QRHX-MS group presented a significant increase in the number of autophagosomes and autolysosomes (*p* < 0.001), which was comparable to that in the RAPA group. The number of autophagosomes in the 20% QRHX-MS + CQ group was significantly greater than that in the 20% QRHX-MS group (*p* < 0.001), whereas the number of autolysosomes was significantly lower (*p* < 0.001) (Figures 6B, O).

These findings indicate that QRHX-MS increases autophagic flux in H/R-exposed H9c2 cells to decrease CHOP-mediated ER stress-related apoptosis.

### 3.7 QRHX-MS attenuates H/R-induced cardiomyocyte injury, ER stress and apoptosis by increasing FAM134B-driven ER-phagy

**FIGURE 7:**
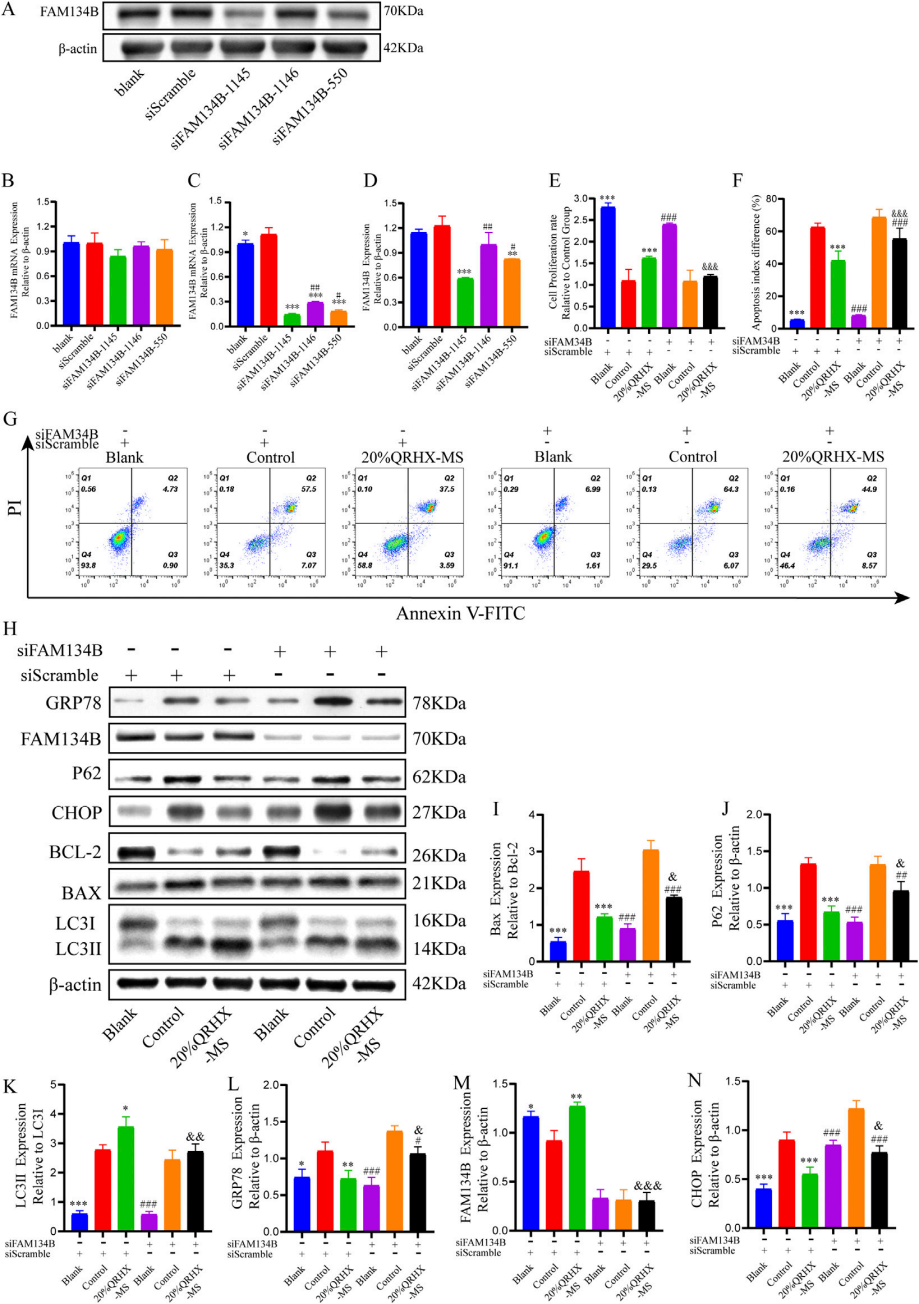
QRHX-MS activates FAM134B-mediated ER-phagy to mitigate ER stress and ER stress-related apoptosis in H/R-exposed cardiomyocytes. **(A, D)** The protein expression of FAM134B after transfection with each siRNA at a concentration of 200 pmol (*n* = 3). **(B–C)** mRNA expression levels of FAM134B in the groups transfected with different siRNAs at concentrations of 100 and 200 pmol (*n* = 6). ^*^ *p* < 0.05, ^**^ *p* < 0.01, and ^***^ *p* < 0.001 in comparison to siScramble and ^#^ *p* < 0.05 and ^##^ *p* < 0.01 in comparison to siFAM134B-1145. **(E)** The viability of cardiomyocytes in the various groups was compared via a CCK-8 assay (*n* = 6). **(F–G)** Flow cytometry was used to assess the percentage of apoptotic cardiomyocytes in the different groups (*n* = 4). **(H)** Protein extracts from cardiomyocytes in each group were analyzed via Western blotting to measure the expression of proteins related to autophagy, ER stress, and apoptosis. **(I–N)** Quantification of the Bax/Bcl-2 and LC3II/LC3I ratios and P62, GRP78, FAM134B, and CHOP levels (*n* = 3). ^*^ *p* < 0.05, ^**^ *p* < 0.01, and ^***^ *p* < 0.001 compared with the siScramble + control group; ^#^ *p* < 0.05, ^##^ *p* < 0.01, and ^###^ *p* < 0.001 compared with the siFAM134B + control group; and ^&^ *p* < 0.05, ^&&^ *p* < 0.01, and ^&&&^ *p* < 0.001 compared with the siScramble + 20%QRHX-MS group. All the data are presented as the means ± SDs.

Initially, when 100 pmol siRNA was used for transfection, there was no significant difference in the level of FAM134B gene expression among the groups transfected with the three different FAM134B siRNAs (Figure 7B). When 200 pmol of siRNA was used for transfection, all three FAM134B siRNAs significantly inhibited FAM134B expression, with siFAM134-1,145 exhibiting the strongest effect (*p* < 0.05) (Figures 7A, C, D). Therefore, cells transfected with 200 pmol siFAM134-1,145 were selected for knockdown experiments.

Compared with the siFAM134B + 20%QRHX-MS group, the siScramble+20%QRHX-MS group presented a markedly increased proliferation rate (*p* < 0.001). Compared with the siFAM134B + 20%QRHX-MS group, the siScramble+20%QRHX-MS group exhibited a significantly reduced proportion of apoptotic cells (*p* < 0.001) (Figures 7E–G). Transfection of siFAM34B partially reversed the QRHX-MS-induced increase in the LC3II/LC3I ratio and Bcl-2 protein levels, as well as the QRHX-MS-induced decrease in GRP78, CHOP, P62, and BAX expression, in H/R-exposed cardiomyocytes (Figures 7H–N).

**FIGURE 8:**
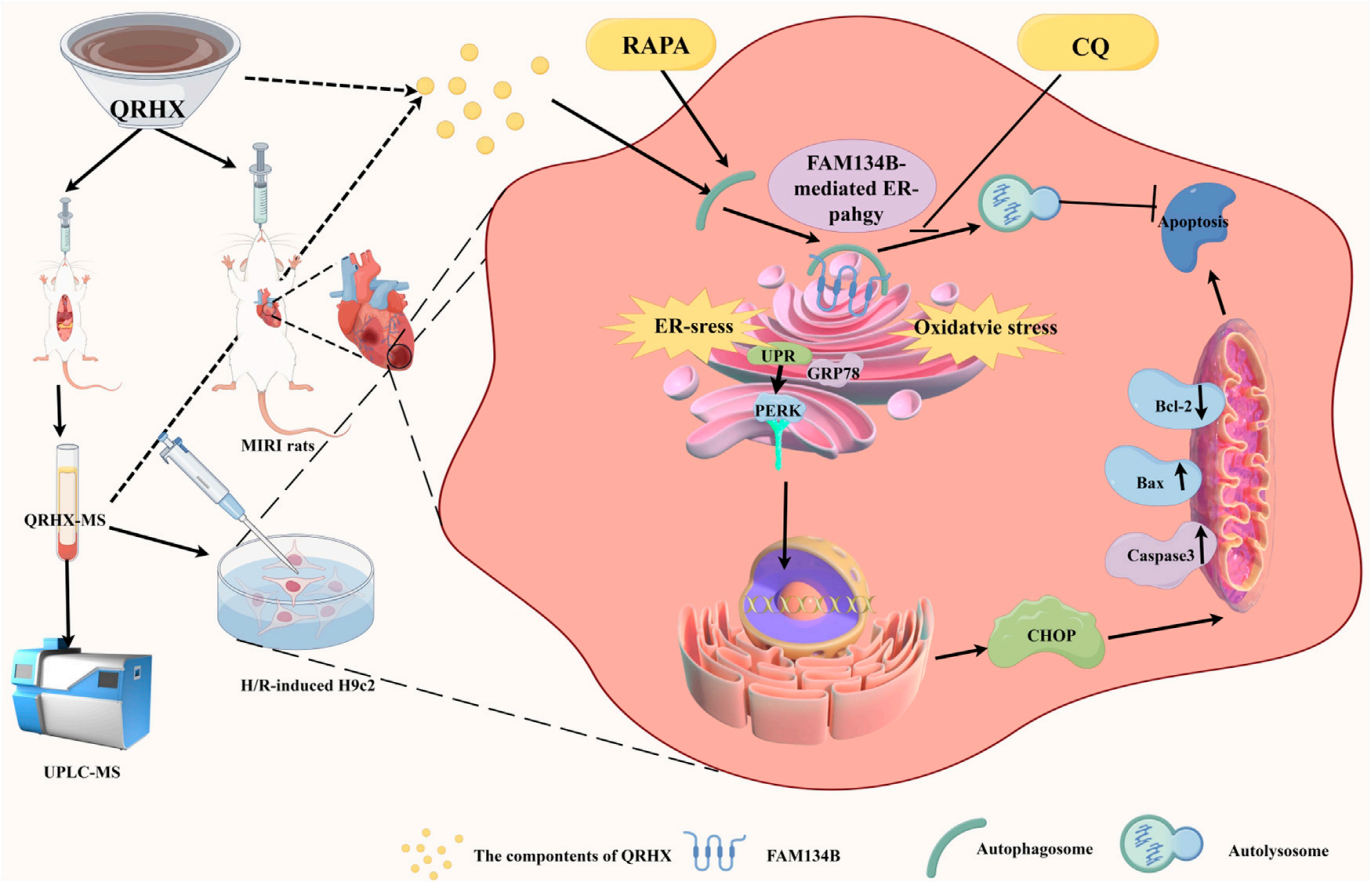
The mechanism by which QRHX alleviates MIRI. QRHX promotes FAM134B-mediated ER-phagy to restrain ER stress, accelerate the degradation of the injured ER, inhibit ER-related apoptosis and oxidative stress, and increase myocardial survival after MIRI.

These results demonstrate that QRHX-MS alleviates cardiomyocyte injury by inhibiting ER stress and CHOP-mediated apoptosis through FAM134B-driven ER-phagy (Figure 8).

## 4 Discussion

Timely reperfusion is the most effective therapy for AMI. However, revascularization of occluded or severely stenotic coronary arteries is often accompanied by MIRI, which exacerbates myocardial injury and even increases the infarct size (Li et al., 2015; Ma et al., 2012; Wu et al., 2021). The pathophysiological mechanisms of MIRI are complex and include oxidative stress, energy metabolism disorders, calcium overload, microcirculation disorders, ER stress, apoptosis, autophagy, and ferroptosis (Heusch, 2020; Zheng et al., 2020). QRHX, a Chinese medicine formula, was developed as a treatment for CHD on the basis of the TCM theory that the pathogenesis of the disease is “heat-toxin-blood stasis”. It has been widely used in clinical practice for more than 20 years and has recently been proven to be effective in treating chest pain associated with CHD, specifically for a “burning sensation in the chest” and “blood stagnation-forming clots” (Jin et al., 2023; Jin et al., 2021; Zuo et al., 2021). Scutellaria baicalensis Georgi (*Huangqin*) and Ilex pubescens Hook. and Arn (*Maodongqing*), components of QRHX, clear heat, dry dampness, and detoxify while promoting blood circulation; Salvia miltiorrhiza Bunge (*Danshen*)*,* Paeonia veitchii Lynch (*Chishao*)*,* Oreocome striata (DC.) Pimenov and Kljuykov (*Chuanxiong*), and Carthamus tinctorius L. (*Honghua*) activate blood circulation and resolve stasis; and Dalbergia odorifera T.C.Chen (*Jiangxiang*) helps to regulate qi, activate blood circulation and relieve pain.

### 4.1 QRHX protects the myocardium from MIRI both *in vivo* and *in vitro*

During MIRI, there is endothelial injury to microvessels, swelling and degeneration of cardiomyocytes, breakage of myocardial myofilaments and infiltration of inflammatory cells, which are directly related to the accumulation of proapoptotic proteins and degradation of antiapoptotic proteins (Heusch, 2020; Xie et al., 2021). The present research demonstrated that QRHX dose-dependently decreased the myocardial infarct size; improved cardiac function; decreased the levels of myocardial enzymes, oxidative stress and apoptosis; and blocked the activity of caspase-3 and Bax/Bcl-2. Additionally, QRHX-H and 20% QRHX-MS had effects in ameliorating MIRI- or H/R-induced myocardial injury similar to those of RAPA, whereas CQ partially reversed the effects of QRHX, suggesting that activating autophagy may be a key mechanism by which QRHX exerts its cardioprotective effects.

### 4.2 UPLC‒MS analysis of the pharmacological metabolites of QRHX-MS

QRHX-MS was found to have 20 main pharmacologically active metabolites, including baicalin, succinic acid, baicalein, cryptotanshinone, isoferulic acid, 3,4-dihydroxybenzaldehyde, salvianolic acid G, caffeic acid, and isocarthamidin, according to UPLC‒MS analysis. Relevant studies have shown that baicalin ameliorates myocardial injury and improves microvascular endothelial function in MIRI rats by promoting the M2 polarization of myocardial macrophages via inhibiting JAK/STAT signaling, increasing the production of nitric oxide and cGMP via the PI3K/AKT/eNOS pathway, regulating PI3K/Akt/NF-κB signaling to facilitate anti-inflammatory and antioxidative processes, and inhibiting ER stress (Bai et al., 2019; Luan et al., 2019; Xu et al., 2020). 3,4-Dihydroxybenzaldehyde alleviates myocardial injury in MIRI rats by regulating NF-κB signaling and inhibiting inflammatory reactions (Wei et al., 2013). Cryptotanshinone exerts cardioprotective effects by reducing microcirculatory disturbances via the suppression of inflammatory cytokines (TNF-α, IL-1β, and IL-6), the inhibition of neutrophil aggregation through the upregulation of MAPK3, and the blockade of the expression of adhesion molecules (VCAM-1 and ICAM-1) via NF‒kB pathway inhibition upon MIRI (Jin et al., 2009; Wang et al., 2021). Caffeic acid and its derivative ameliorate MIRI by mitigating oxidative stress, the inflammatory response, necroptosis, and apoptosis while enhancing myocardial energy metabolism via the SIRT1/eNOS/NF-κB and AMPK/Akt/HO-1 pathways (Ku et al., 2016; Lee et al., 2015; Li D. et al., 2018). These results imply that the primary metabolites of QRHX-MS underlie the pharmacological effect of QRHX in alleviating MIRI.

### 4.3 QRHX promotes myocardial autophagic flux to negatively regulate ER stress in MIRI

Autophagy, triggered by stress, leads to the removal of excessive or abnormal proteins, cytoplasmic components, or damaged organelles within autolysosomes, which is crucial for cell survival. Research has revealed that autophagy provides protection against myocardial ischemia but worsens myocardial damage during reperfusion (Sciarretta et al., 2018). However, Ma et al. (2012) reported that although the levels of Beclin-1 and LC3II are increased in the ischemic myocardium, indicating the promotion of autophagosome formation, autolysosomal degradation is hindered, as indicated by elevated levels of P62. Impaired autophagic flux leads to autophagosome accumulation, increased reactive oxygen species (ROS) production, and exacerbation of myocardial injury during MIRI (Song et al., 2018). Xie et al. (2021), Xie et al. (2014) demonstrated that the inhibition of histone deacetylase activators activates autophagic flux in the border zone of the infarcted myocardium in the early stage of MIRI and suppresses ROS generation, thereby significantly reducing the myocardial infarct size by 40% and ultimately improving myocardial contractile function.

The ER is an important membrane-bound organelle involved in protein, lipid, and cholesterol synthesis as well as calcium ion storage in eukaryotic cells. ER stress is triggered upon MIRI. The primary manifestation of ER stress is the unfolded protein response (UPR), which initially acts as a survival mechanism by decreasing the accumulation of unfolded proteins to maintain ER homeostasis. However, when ER stress increases greatly and leads to cellular damage, related apoptotic pathways, such as the PERK‒CHOP pathway, are activated to eliminate impaired cells (Song et al., 2017; Song et al., 2018). Activation of the PERK‒CHOP pathway leads to the upregulation of Tribbles-related protein 3, increases the expression of the proapoptotic proteins Bax and Bak, suppresses the expression of the antiapoptotic protein Bcl-2, and ultimately facilitates cellular apoptosis (Fu et al., 2010; He et al., 2021). Yu et al. (2015) and Han et al. (2019) have shown that melatonin, by activating melatonin receptor 1b or the SIRT1 signaling pathway, inhibits ER stress, CHOP, and caspase-12-mediated apoptotic pathways, significantly alleviating MIRI-induced myocardial apoptosis, infarct expansion, and cardiac dysfunction.

Autophagy, as a protective negative feedback mechanism, alleviates excessive ER stress by degrading unfolded or misfolded proteins and the damaged ER, exerting a protective effect on cells under stress (Biczo et al., 2018; Kokten et al., 2018; Li X. et al., 2018; Wang et al., 2016). Li X. et al. (2018) reported that renal ischemia‒reperfusion leads to increased cell apoptosis, ER stress and autophagy. Pretreatment with RAPA further promotes early autophagic flux, significantly reducing ER stress and renal tubular cell apoptosis. Guan et al. (2019) demonstrated that increasing autophagic flux in H9c2 cardiomyocytes exposed to H/R decreases cellular apoptosis by inhibiting ER stress.

In the present study, QRHX increased the colocalization of LAMP2 and LC3B in a dose-dependent manner, whereas 20% QRHX-MS effectively increased autophagic flux in H/R-exposed cardiomyocytes. Furthermore, compared with that in the sham group and blank group, the expression of LC3II/LC3I, P62, GRP78, PERK, CHOP, and Bax/Bcl-2 in the MIRI group and H/R group was notably greater, suggesting that disruption of autophagic flux, activation of ER stress and apoptosis are involved in MIRI. QRHX and QRHX-MS further increased the LC3II/LC3I ratio but downregulated P62 in cardiomyocytes subjected to MIRI, indicating the promotion of autophagic flux, which also decreased GRP78, PERK, CHOP, and caspase3 levels and the Bax/Bcl-2 ratio, revealing the inhibition of ER stress and ER stress-related apoptosis. The beneficial effects of QRHX were similar to those of RAPA but were partially counteracted by CQ. These results confirm that QRHX protects the myocardium by increasing autophagic flux to inhibit ER stress and the activation of related apoptotic pathways in MIRI.

### 4.4 QRHX regulates the autophagy-ER stress axis through FAM134B-mediated ER-phagy in MIRI

There is no consensus about the impact of FAM134B-mediated ER-phagy on cardiomyocytes. On the one hand, Yang et al. (2022) demonstrated that FAM134B-mediated ER-phagy triggered by apelin-13 induces myocardial hypertrophy through activation of the pannexin-1/eATP/P2X7 axis, whereas inhibition of FAM134B-mediated ER-phagy alleviates apelin-13-induced cardiac hypertrophy. Qu et al. (2022) reported that doxorubicin induces the formation of multiple pores on the ER membrane via the caspase11/gasdermin D pathway, leading to ER stress, the activation of FAM134B-mediated ER-phagy, and subsequent myocardial apoptosis and pyroptosis, thereby causing drug-related cardiac toxicity. Preetha Rani et al. (2022) demonstrated that palmitic acid inhibits SEC62 and RTN3 expression while upregulating FAM134B expression to inhibit ER-phagy and thus relieve ER stress and apoptosis mediated by CHOP, caspase-12, and calreticulin in the myocardium of diabetic rats.

On the other hand, Li et al. (2021) reported that RAPA restrains inflammatory infiltration and proapoptotic protein expression in the myocardium in sepsis by promoting FAM134B-mediated ER-phagy and that the cardioprotective effect of RAPA is reversed by knocking out FAM134B. Zhang et al. (2020) demonstrated that adiponectin suppresses ER stress and CHOP-mediated apoptosis in chronic intermittent hypoxia-exposed cardiomyocytes by activating the AMPK pathway to increase SEC62-mediated ER-phagy.

In the present study, QRHX increased the expression of FAM134B, the numbers of ER-phagosomes and autolysosomes in a dose-dependent manner in cardiomyocytes upon MIRI. Compared with RAPA, QRHX more effectively stimulated the colocalization of FAM134B and LC3B, and this effect was partially reversed by the autophagic flux inhibitor CQ. Interestingly, after FAM134B was knocked down in H9c2 cells, the ability of QRHX to stimulate autophagic flux and alleviate ER stress and apoptosis was partially counteracted, indicating that FAM134B-driven ER-phagy is an important mechanism through which QRHX regulates the autophagy‒ER stress axis to ameliorate MIRI.

## 5 Limitations

Our research has certain limitations. For example, our studies on MIRI were not conducted in animal models with CHD risk factors, such as diabetic or hyperlipidemic rats, which weakens the clinical translational potential of our research. It is believed that further research on MIRI in the context of comorbid conditions will provide a more solid molecular biological basis for the extensive clinical application of QRHX.

## 6 Conclusion

QRHX may be an important adjunct medicine used for alleviating myocardial injury, apoptosis and infarct size expansion in MIRI by regulating the autophagy‒ER‒stress axis via FAM134B-mediated ER-phagy.

## Data Availability

The original contributions presented in the study are included in the article/Supplementary Material, further inquiries can be directed to the corresponding authors.
